# Investigation of Biaxial Properties of CFRP with the Novel-Designed Cruciform Specimens

**DOI:** 10.3390/ma15197034

**Published:** 2022-10-10

**Authors:** Xiaowen Zhang, Haiyang Zhu, Zhixing Lv, Xiangrun Zhao, Junwei Wang, Qi Wang

**Affiliations:** 1 School of Materials Science and Engineering, Dalian University of Technology, Dalian 116024, China; 2 National Key Laboratory of Combustion, Thermo-Structure and Flow, The 41st Institute of the Fourth Academy of CASC, Xi’an 710025, China; 3 China Safety Technology Research Academy, Beijing 100053, China; 4 Xi’an North Qinghua Electromechanical Co., Ltd., Xi’an 710025, China; 5 Dalian Yuchen High Tech Material Co., Ltd., Dalian 116023, China; 6 School of Electrical and Electronic Engineering, Nanyang Technological University, 50 Nanyang Avenue, Singapore 639798, Singapore

**Keywords:** carbon-fiber-reinforced polymer, biaxial testing, finite element analysis, failure analysis

## Abstract

The biaxial loading properties of carbon-fiber-reinforced polymer (CFRP) are critical for evaluating the performance of composite structures under the complex stress state. There are currently no standardized specimens for the CFRP biaxial experiments. This work developed a new design criterion for the cruciform specimen coupled with the Hashin criterion. The finite element analysis was conducted to investigate the effect of geometric parameters on the stress distribution in the test area. The embedded continuous laying method (ECLM) was proposed to achieve the thinning of the center of the test region without introducing defects. The manufacturing quality of the cruciform specimens was verified by the ultrasonic C-scanning test. The biaxial test platform consisting of the biaxial loading system, digital image correlation (DIC) system, strain electrical measurement system, and acoustic emission detection system was constructed. The biaxial tensile tests under different biaxial loading ratios were conducted. The results showed that the biaxial failure efficiently occurred in the test area of the cruciform specimens designed and manufactured in this paper. The failure modes and morphology were characterized using macro/microscopic experimental techniques. The biaxial failure envelope was obtained. The results can be used to guide the design of composite structures under biaxial stress.

## 1. Introduction

Carbon-fiber-reinforced polymer (CFRP) composites are widely used in various fields, especially in aerospace equipment, such as plate skins, and pressure vessels in aerospace equipment [1,2,3,4]. The reliable application of composites depends on the accurate evaluation and prediction of their mechanical properties [5,6]. Numerous studies have used uniaxial tests to identify the properties of composites, contributing to a wider application of CFRP [4,7,8,9,10,11,12]. However, the uniaxial mechanical tests are limited in characterizing the real mechanical responses of composites under the complex loading state in engineering applications, for example, the filament wound pressure vessels (FWPV) [13,14,15,16]. Therefore, the biaxial test is significant for identifying the strength of composites under actual working conditions [17,18,19,20,21,22,23].

The design of the biaxial specimen is critical in obtaining reliable biaxial testing results that satisfy the requirements of [24] uniform biaxial stress state appearing in the effective test area, initial failure arising in the region of the biaxial stress state, obtaining the stress state of the effective test area directly, and controlling each stress component in the biaxial load independently [24]. According to the above requirements, the cruciform specimen is most adopted among the different designs of composite biaxial specimens due to their simplicity in varying the biaxial stress state. Four loading arms and four actuators are clamped to transfer the biaxial loadings to the central test area of the cruciform specimen during the loading process, making the load ratio of the two directions adjustable.

However, severe stress concentration can easily occur at the corner between the two adjacent loading arms during the loading process of cruciform biaxial specimens. The stress concentration can result in premature tearing of the end of the loading arms, which causes inaccurate test results or test failures.

Smits et al. [25] proposed that the stress concentration should be weakened outside the effective test area and the shear stress should be weakened in the effective test area for the design of cruciform biaxial specimens, so as to ensure that the initial failure occurs in the effective test area [25]. Moreno et al. [26] discussed the influence of the geometry parameters of cruciform specimens on the initial failure region, including the width of loading arms and the side length of the effective test area. They found that correct failure modes in the typical cruciform specimens were difficult to achieve. Welsh et al. [27] designed a cruciform biaxial specimen, whose initial failure occurs in the effective test area by optimizing the shape and the thickness of the effective test area. Makris et al. [28] proposed an optimization design method for biaxial specimens which aims at the ratio of the standard deviation and the mean value of the principal strain in the test area. Gower et al. [29] designed a thinned circular effective test area to avoid the stress concentration at the corner of specimens.

Thinning of the central test area is demonstrated as a valid strategy to reduce the stress concentration of the corners, whose most common processing method is milling. Although milling has the advantage of low cost and simple processing, it leads to fiber discontinuity and unexpected delamination in the variable thickness region. Accordingly, the possibility of failure in the variable thickness region is greatly amplified due to the initial defects in the specimen caused by the milling process [30,31]. Lamkanfi et al. [32] discussed the effect of geometric discontinuities in the milling area on the strain distribution uniformity of the effective test area by measuring the strain distribution with the digital image correlation (DIC) technique. The results show that the stress concentration in the milling area and the variable thickness region are caused by the initial cracks in the transition area produced during milling, which is independent of load direction and load type of biaxial tensile. The experimental phenomena are explicated by the elasticity solution of inter-laminar stresses in composite laminates [33]. Correa et al. [34] and Moreno et al. [26] attempted the approach of pasting open glass fiber-reinforced composite plates or metal plates on the variable thickness region to strengthen the milling edges. However, it failed to achieve the arbitrary loading ratio or equivalent stiffness of arms in two directions. Therefore, it is crucial to realize the uniform stress transition in the effective test region during the biaxial loading process, to avoid the premature failure of the variable thickness region.

In this paper, the design criteria of the cruciform specimen are developed by introducing the Hashin criterion to ensure that the failure occurs in the central testing region of the specimen. A new laying method called the embedded continuous laying method is proposed for the cruciform specimen. Then, the cruciform specimens are manufactured and tested by the acoustic emission test to investigate the internal defects. The biaxial test platform consisting of the biaxial loading test system, three-dimensional strain optical measurement system, strain electrical measurement system, and acoustic emission detection system is constructed. During the biaxial test, strain gauges and digital image correlation (DIC) are utilized to capture the strain evolution of the specimens. Meanwhile, the acoustic emission is used to identify the failure modes of the specimens. The biaxial tensile tests under different loading ratios in the X and Y direction are carried out, and the variations of strain fields and the acoustic emission signals during the loading are monitored. Utilizing the cruciform specimen designed in this paper, combined with constructed biaxial test platform and micromechanical analysis, the biaxial failure mechanism and the failure envelope of CFRP are effectively obtained.

## 2. Design of the Cruciform Specimen

A simulation-driven specimen design method is proposed in this section to ensure that failures generated under the biaxial loading occur within the effective test region. The design criterion for the cruciform specimen is developed to identify the design requirements. Then, the design parameters are characterized by numerical simulation.

### 2.1. Design Criterion

As mentioned, the ideal specimen for the biaxial test should have a smaller stress concentration factor and a higher degree of stress uniformity in the effective test region. The location of initial failure in cruciform specimens can be determined by the stress concentration factor $F_{r}$, which is expressed as [18]:

(1) $$F_{r}=\frac{e_{\mathrm{eff}}^{2}}{e_{\mathrm{load}}^{2}}$$

where $e_{\mathrm{eff}}$ and $e_{\mathrm{load}}$ are the strength discriminant factors in the effective test region and biaxial loading region, respectively. Hereby, the Hashin criterion [35] has been adopted to calculate $e_{\mathrm{eff}}$ and $e_{\mathrm{load}}$ as:

(2) $$e_{\mathrm{eff}}=\sqrt{\mathrm{Max}{\left(\frac{\sigma_{\mathrm{eff}}^{f}}{S_{f}}\right)}^{2}+\mathrm{Max}{\left(\frac{\tau_{\mathrm{eff}}^{}}{S_{C}}\right)}^{2}}$$

(3) $$e_{\mathrm{load}}^{}=\sqrt{\mathrm{Max}{\left(\frac{\sigma_{\mathrm{load}}^{f}}{S_{1}}\right)}^{2}+\mathrm{Max}{\left(\frac{\tau_{\mathrm{load}}^{}}{S_{C}}\right)}^{2}}$$

where $\sigma_{\mathrm{eff}}$ and $\tau_{\mathrm{eff}}^{}$ are the longitudinal stress and the in-plane shear stress in the effective test region of the specimens, respectively. $\sigma_{\mathrm{load}}^{f}$ and $\tau_{\mathrm{load}}^{}$ are the longitudinal stress and the in-plane shear stress of the loading arms in the biaxial cruciform specimens, respectively. $S_{f}^{}$ and $S_{C}^{}$ are the longitudinal tensile strength and the in-plane shear strength of the composites, respectively. The smaller $F_{r}$ means the failures are easier to occur in the effective test region.

The degree of stress uniformity in the effective test region of the cruciform specimen under biaxial tensile loadings is determined by the stress variation coefficient δ, given as:

(4) $$\delta =\frac{D_{e_{\mathrm{eff}}^{}}}{{\bar{e}}_{\mathrm{eff}}^{}}$$

where $D_{e_{\mathrm{eff}}}$ and ${\bar{e}}_{\mathrm{eff}}$ are the standard deviation and mean value of strength discriminant factor $e_{\mathrm{eff}}$ in the effective test region, respectively.

A weighted design function $f\left(F_{r},\delta \right)$ is established for the combination of stress concentration factor and stress variation coefficient, expressed as:

(5) $$f\left(F_{r},\delta \right)=\frac{1}{wF_{r}+\left(1-w\right)\delta }$$

where w is the weighting factor, set as 0.8 [29]. $f\left(F_{r},\delta \right)$ is directly related to stress distribution uniformity. $f\left(F_{r},\delta \right)$ is empirically equated to a minimum value of 2.3 to ensure the cruciform specimen fulfills the request of homogenous failure.

### 2.2. Geometric Parameter Characterization

#### 2.2.1. Geometric Configuration and Parameters

**Figure 1:**
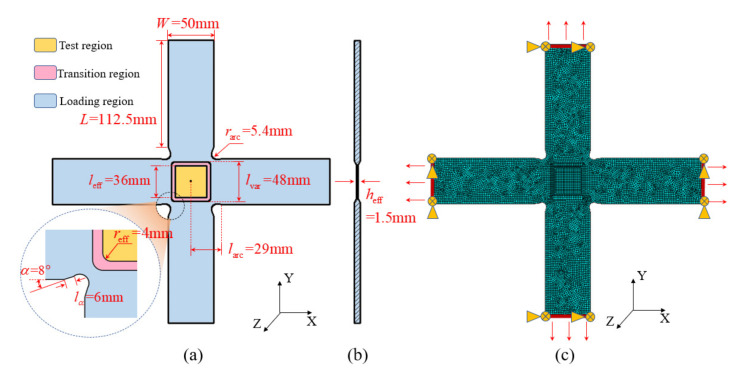
Initial configuration design of the CFRP biaxial specimens. (**a**) Geometric parameters, (**b**) side view of the specimen, (**c**) finite element model of the specimen.

Figure 1a presents the initial configuration design of the CFRP biaxial specimens, whose specific geometric parameters are listed in Table 1. The effective test region is thinned to ensure that the initial failure occurs herein. The uniform thickness gradient is adopted in the transition region, which is between the loading arms and the effective test region. The tapered chamfer is used to transmit stresses to the center of the cruciform specimens. The fillets are added in the transition region between the two adjacent loading arms to reduce the stress concentration. A necking angle is used at the end of each loading arm, which is close to the fillet to reduce the bearing area of the effective test region.

The material system used in this paper is T300/Epoxy, whose mechanical properties are listed in Table 2. The stiffness ratio of the specimens in the X and Y direction is 2:1 to simulate the typical pressure vessel wall under uniform internal pressure. Based on the uniform and symmetrical laying principle, the ply sequence of the effective test region is designed as [90°/0°_2_]_2s_.

The finite element model for the cruciform specimen is established in the commercial finite element analysis (FEA) software ABAQUS with eight-node hexahedral incompatible CSD8I elements, as shown in Figure 1c. The displacement constraints in Y and Z directions are applied to the ends of the X-directional loading arms, the displacement constraints in X and Z directions are applied to the ends of the Y-directional loading arms, and the uniform loadings are applied to the ends of the four loading arms.

#### 2.2.2. Effect of Geometric Parameters on the Stress Distribution

**Figure 2:**
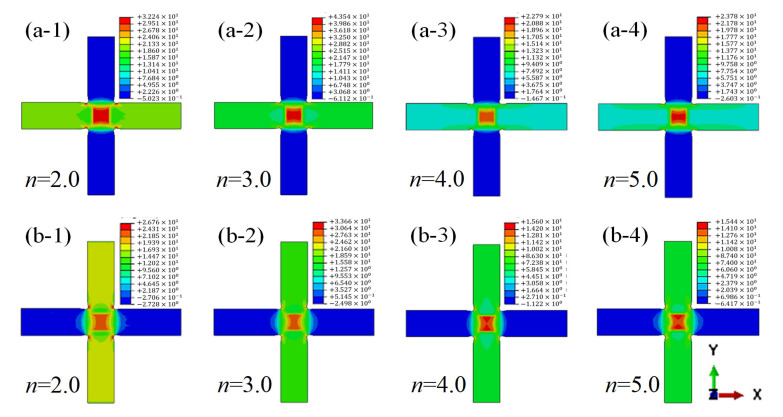
Maximum principal stresses distribution of cruciform specimen (**a**) stresses in 0° ply when *n* set as 2.0, 3.0, 4.0, and 5.0. (**b**) Stresses in 90° ply when *n* set as 2.0, 3.0, 4.0, and 5.0.

Different loading arm thicknesses are set to investigate the effect of thickness of the loading arms on the stress distribution in the cruciform specimen. The loading arm thicknesses of 3.0 mm, 4.5 mm, 6.0 mm, and 7.5 mm are considered in this section. Each of their thickness are 2.0, 3.0, 4.0 and 5.0 times of the thickness of effective test region. The ratio of the thickness of loading arms and effective test region are defined as *n*. The stress distribution of the specimens under biaxial tension with the loading ratio of 2:1 in X and Y directions is analyzed. From the results shown in Figure 2, when *n* is set to 2.0 or 3.0, apparent stress concentration occurs at the corner of the loading arms adjacent to the 0° and 90° plys. When *n* equals 4.0 or 5.0, the maximum principal stress of the laminate occurs in the central test area, and the stress concentration at the corner of the loading arm is restrained, as shown in Figure 2b.

**Figure 3:**
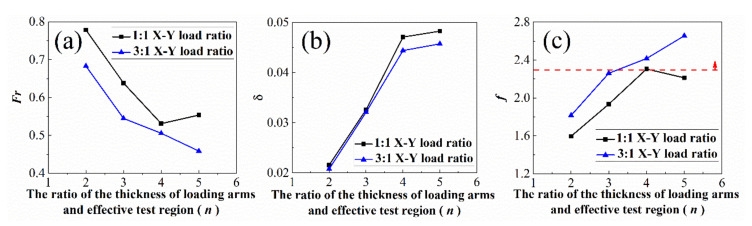
The relationship between (**a**) the stress concentration factor, (**b**) the stress variation coefficient, and (**c**) the weighted design function and the ratio of the thickness of loading arms and effective test region. The red line presents the design critical value $f_{cri}$.

Figure 3 illustrates the relationship between the stress concentration factors, stress variation coefficients, and the variable *n* (the ratio of the thickness of the loading arms to the thickness of the effective test region). To select the appropriate value of *n*, the stress concentration factors $F_{r}$ and the stress variation coefficient δ are employed as the evaluation index, and an evaluation criterion $f\left(F_{r},\delta \right)$ is further proposed. The values of $F_{r}$, δ, and $f\left(F_{r},\delta \right)$ are calculated and shown in Figure 3 when *n* takes the value of 2.0, 3.0, 4.0 and 5.0. Figure 3a shows that with the increase in *n*, $F_{r}$ decreases monotonously when the X-Y loading ratio is 3:1. This indicates that a larger thickness ratio of loading arms and the effective test region will result in a smaller stress concentration. While the loading ratio is 1:1, the relationship is not monotonous. $F_{r}$ increases when *n* equals 5.0. This is because when *n* increases to a certain value, the maximum shearing stress will extend to the transition region because of the thin effective test region. In the case of 1:1 loading ratio, the stress concentration is more sensitive to the change of $e_{eff}$ value caused by the thinning of the test region. Figure 3b shows that, for both cases of 1:1 and 3:1 loading ratios, δ increases with the increase in *n*. When the value of *n* reaches 4.0, the slope is clearly slowing down. The results indicate that a larger thickness ratio of loading arms and the effective test region will cause a more even stress distribution. However, the effect is limited when *n* reaches 4.0. The results of the criterion $f\left(F_{r},\delta \right)$ is shown in Figure 3c. It can be seen that $f\left(F_{r},\delta \right)$ increases monotonously when the X-Y loading ratio is 3:1, while $f\left(F_{r},\delta \right)$ increases first then decreases with the inflection point of *n* = 4 for the case of 1:1 loading ratio. With consideration of that $f\left(F_{r},\delta \right)$ should be higher than 2.3 based on the discussion in Section 2, *n* = 4 is selected to design the thickness of the cruciform specimen, for which the thickness of the effective test region is 8.0 mm.

**Figure 4:**
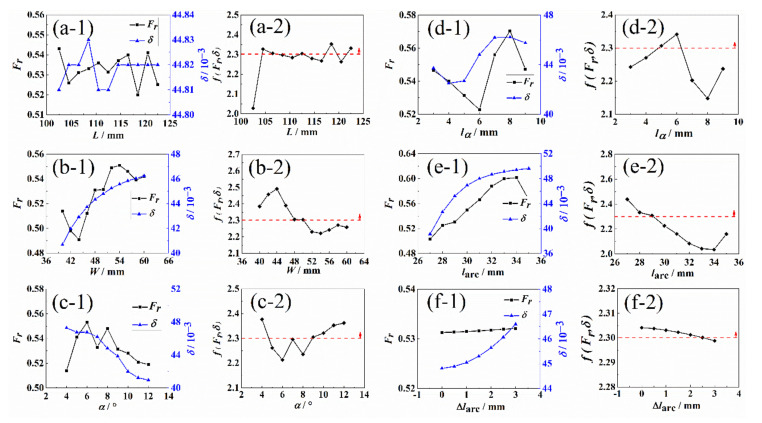
The relationship between the (**a-1**–**f-1**) stress concentration factor $F_{r}$, stress variation coefficient δ and (**a-2**–**f-2**) the weighted design function $f\left(F_{r},\delta \right)$ and (**a**) the length of loading arms L, (**b**) The width of loading arms W, (**c**) The necking angles α, (**d**) the distance between necking angle and transition area $l_{\alpha }$, (**e**) center region of transition arc $l_{arc}$, (**f**) eccentricity of the effective test region $\Delta l_{c}$. The red dash line and arrow indicate the allowable scope.

Similarly, the relationship between the length of loading arm L, the width of loading arm W, the necking angle α, the distance between necking angle and transition area $l_{\alpha }$, the distance between the center of transition arc and the center of cruciform specimen $l_{\mathrm{arc}}$, and the eccentricity of effective test region $\Delta l_{c}$ with the stress concentration factor $F_{r}$, and stress variation coefficient δ are shown in Figure 4. The above geometric parameters are assumed to be independent. With the change of most of the geometric parameters, as shown in Figure 4a–e, the tendencies of $F_{r}$ and δ are non-monotonic, leading to $f\left(F_{r},\delta \right)$ having multi-peak values. In Figure 4f, $F_{r}$ and δ vary monotonously with the change of $\Delta l_{c}$, which indicates that the small eccentricity of the effective test region is beneficial to the uniformity of stress in the test area. During the manufacturing process of the specimens, the process accuracy of the above parameters should be controlled within the allowable scope as marked in Figure 4 to avoid unexpected specimen failure caused by machining error or process-induced distortions [36,37].

## 3. Specimen Manufacturing and Experimental Setup

### 3.1. Manufacturing Method

The manufacturing of cruciform specimens usually adopts the conventional lay-up procedure combined with the cutting and milling process, whose processing is as follows:

(a) Prepare the required number of layers prepregs and cut them into the shape of the cruciform specimen.
(b) Lay the prepregs on the female mold in the pre-defined layup sequence and close the male mold.
(c) Heat the mold under the manufacturer’s recommended cure cycle and pressure, then demold to obtain the specimens.
(d) Mill the specimens to obtain the transition region and the central test region.

**Figure 5:**
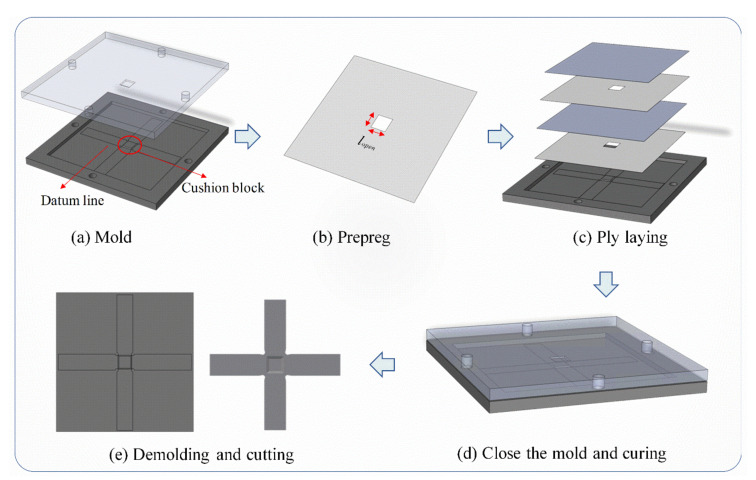
Schematic diagram of the cruciform specimen manufacturing.

It can be found that the outer surfaces of the specimens manufactured by the above method are composed of cut fiber caused by the milling process. The discontinuous fibers in the transition and central test areas will generate interlaminar stress during the biaxial tension. The interlaminar stress may result in early failure near the transition area. To deal with this problem, a novel manufacturing method for the cruciform specimen has been proposed in this section. A new laying method “Embedded Continuous Laying Method” (ECLM) was designed for the cruciform specimen to avoid the initial defect caused by the milling process on the specimen and transfer the stress from the loading arm to the central area more effectively. The schematic diagram of the specimen manufacturing is shown in Figure 5. The specific steps are as follows:

(a) Delimit the edge datum line on the mold. Assemble the metal cushion block with same dimensions of the effective test region on the mold.
(b) Cut the CFRP prepreg into 350 mm × 350 mm. Some of which are cut with different opening sizes in the central area for the realization of variable thickness regions of cruciform specimens. The opening sizes $l_{open}$ of the cruciform specimens are determined by Equation (6).
  (6) $$l_{open}\left\{\begin{array}{cc} =0 & n_{l}=1,8,15,22,23,24\\ =l_{\mathrm{eff}}-n_{l}\left(l_{\mathrm{var}}-l_{\mathrm{eff}}\right)/n_{open} & \;\;\;\;2\leq n_{l}\leq 7\\ =l_{\mathrm{eff}}-\left(n_{l}+1\right)\left(l_{\mathrm{var}}-l_{\mathrm{eff}}\right)/n_{open} & \;\;\;\;9\leq n_{l}\leq 14\\ =l_{\mathrm{eff}}-\left(n_{l}+2\right)\left(l_{\mathrm{var}}-l_{\mathrm{eff}}\right)/n_{open} & \;\;\;\;16\leq n_{l}\leq 21 \end{array}\right.$$
  where $n_{l}$ is the half of the lay number of loading arm, $n_{open}$ is the half of the lay number of opening prepreg.
(c) Lay the cut CFRP prepreg according to the edge datum line with the arranged laying subsequences and opening sizes. The laying subsequences of loading arms and effective test area are [90°/0°_2_]_8s_ and [90°/0°_2_]_2s_, respectively.
(d) Close the mold and cure the CFRP plates by hot press. After curing, cut the cured CFRP plates using computer numerical controlled (CNC) machine to obtain the cruciform specimens.

The specimens manufactured by ECLM will ensure a continuous distribution of fibers, and thus obtain an even distribution and smooth transition of stress. However, the manufacturing cost is relatively higher because more working time is needed.

**Figure 6:**
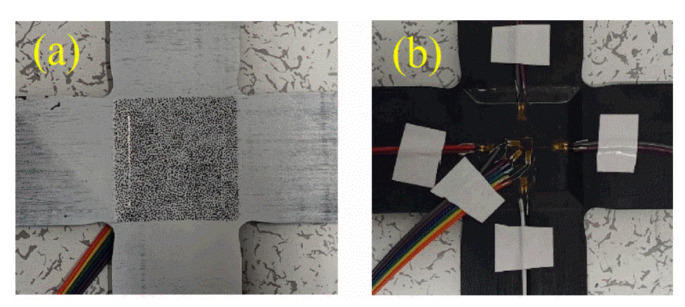
Images of the cruciform specimen. (**a**) With speckles painted, (**b**) with strain gauges pasted.

After manufacturing, the specimens are sprayed with speckles on one side of the effective test region, as shown in Figure 6a. Four uniaxial strain gauges and one triaxial strain gauge are pasted on the other side of the effective test region of the specimen, as shown in Figure 6b.

**Figure 7:**
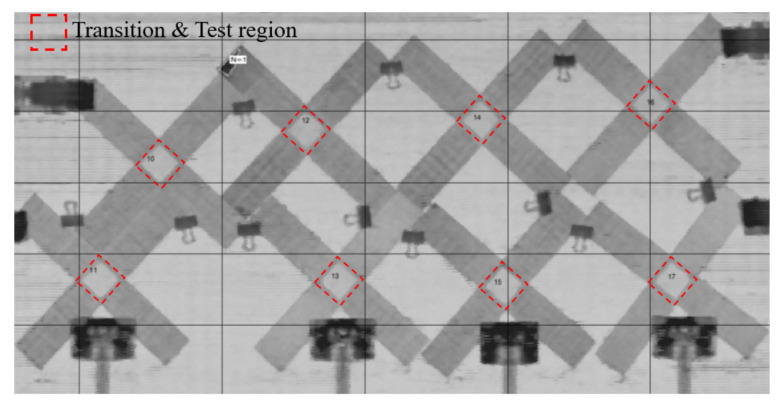
Ultrasonic C-scan results of the manufactured cruciform specimens.

On the other hand, several cruciform specimens with the same geometry are manufactured, of which the transition area is processed by milling to achieve the variable thickness for the comparing purpose. The process quality of the manufactured cruciform specimens was tested by ultrasonic C-scanning (Epoch600 High Speed 10-Axes 3D-NDI ultrasonic scanner). The damages will be recognized as darker areas by ultrasonic C-scanning. It can be seen from Figure 7 that there are no defects such as voids, delamination, and resin distribution unevenness in the effective test regions and transition regions of cruciform specimens. This indicates that the proposed method can ensure the high quality of the cruciform specimens.

### 3.2. Experimental Setup

**Figure 8:**
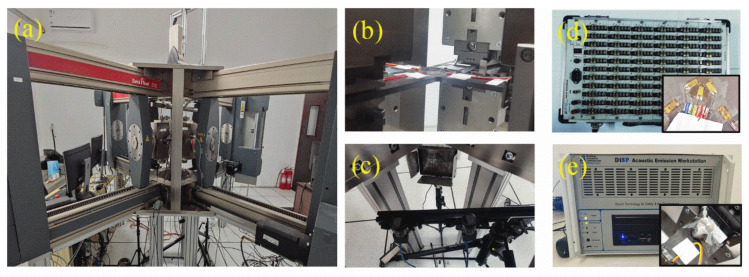
The biaxial test platform. (**a**) Digital biaxial loading system, (**b**) biaxial loading test system, (**c**) three-dimensional strain optical measurement system, (**d**) DH-3820 dynamic strain acquisition system, (**e**) acoustic emission detection system.

The biaxial test platform constructed in this paper is shown in Figure 8. The platform consists of the Zwick-Z150 digital biaxial loading system, the three-dimensional strain optical measurement system, the strain electrical measurement system, and the acoustic emission detection system. The loading system is equipped with a 150 kN force sensor with a load accuracy of 300 N to 1500 N, and a 100 kN spiral wedge tensile fixture. The video extensometer, with a load accuracy of 0.25 μm, is installed in the test facility. During the loading process, the load can be adjusted automatically according to the binding force of the four axes. The wedge fixture is equipped in the biaxial tensions with the function of self-clamping when the actuator moves backward.

During the biaxial tension processing, continuous images of specimens covered with speckles were taken by the industrial camera. Concurrently, the non-contact three-dimensional strain optical measurement system MatchID Stereo, presented as Figure 8c, is used to measure the full-field strain of the specimen by tracking the deformation of the images. Specifically, the speckles distributed on the surface of the specimen are used as the information carrier. The displacement vectors of speckles are tracked and analyzed by digital image correlation (DIC) technique during the loading to determine the global strain field. DH-3820 dynamic strain acquisition system, as shown in Figure 8d, is employed to evaluate the strain accuracy of local points in the effective test region with a 5 Hz sampling frequency.

The acoustic emission probes (PAC, MISTRAS Group, Inc., Princeton Junction, NJ, USA) are arranged near the effective test region on each of the four loading arms. PCI-2 acoustic emission signal acquisition system is used to detect the acoustic emission of the cruciform specimens as shown in Figure 8e. The change of impact amplitude, impact times, energy, and duration during the loading process are also detected for the evaluation of the failure modes and failure severity. The frequency range of the sensor is 50 kHz to 200 kHz, the center frequency is 150 kHz, the preamplifier is 2/4/6 type, the gain value is 20 dB, the sampling set amplitude threshold is 40 dB, the sampling frequency is 1 MHz, the impact time is 150 μs, the impact locking time is 300 μs, and the maximum duration is 100 ms.

## 4. Results and Discussion

### 4.1. Comparison of Different Processing of Cruciform Specimens

**Figure 9:**
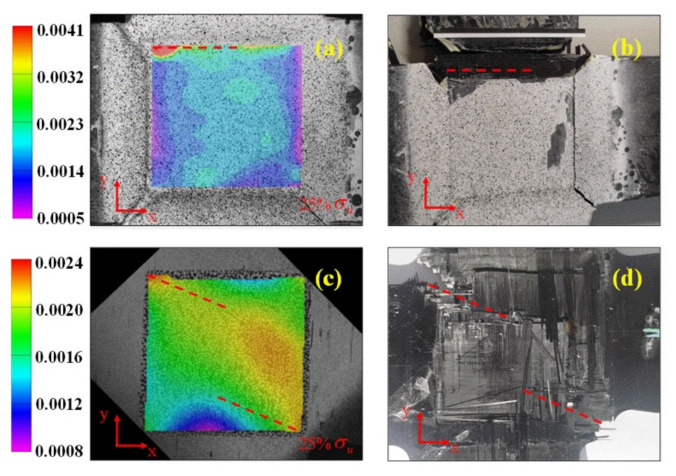
Strain field and failure morphology of the cruciform specimens processed by milling (**a**,**b**) and ECLM (**c**,**d**).

A comparison of the biaxial test results between two sets of cruciform specimens, one manufactured using ECLM and the other processed by milling, was made. The loading ratio in the X and Y direction is 1:1 for both specimens. The difference between the test results of the specimens manufactured by both processes can be found in Figure 9. From the strain field distribution captured by the DIC, there was stress concentration at the edge of the central test area of the latter cruciform specimen (Figure 9a), resulting in the fracture of the loading arm (Figure 9b). Thus, no valid biaxial test data can be obtained. In contrast, the strain distribution is more uniform in the central test area of the former specimen, and the failure occurred in the central test area (Figure 9c,d). The results indicate that the ECLM is more efficient to achieve the thinning of the central test area without introducing defects.

### 4.2. Mechanical Response

(1) Stress–strain curves

**Figure 10:**
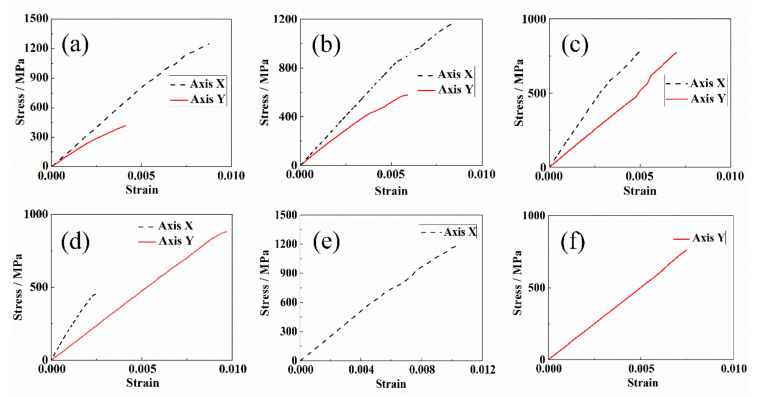
The stress–strain curves with loading ratio in X and Y direction by (**a**) 3:1, (**b**) 2:1, (**c**) 1:1, (**d**) 0.67:1, (**e**) 1:0, (**f**) 0:1.

The biaxial tension tests were carried out in force-controlling mode. The loading rates in the X direction and Y direction are 1 kN/min. The loading ratios of the loading arms in the X and Y direction are 3:1, 2:1, 1:1, 0.67:1, 1:0, and 0:1, where the last two conditions represent the uniaxial loading. Three valid biaxial tension tests were carried out under each loading ratio. The typical stress–strain curves of the biaxial tension with different loading ratios are presented in Figure 10a–f. The results show that there are significant differences in the ultimate strength of the cruciform specimens when the loading ratios are different. This indicates that the biaxial test is of great significance to evaluate the strength properties of composites under complex stress conditions.

(2) DIC results

**Figure 11:**
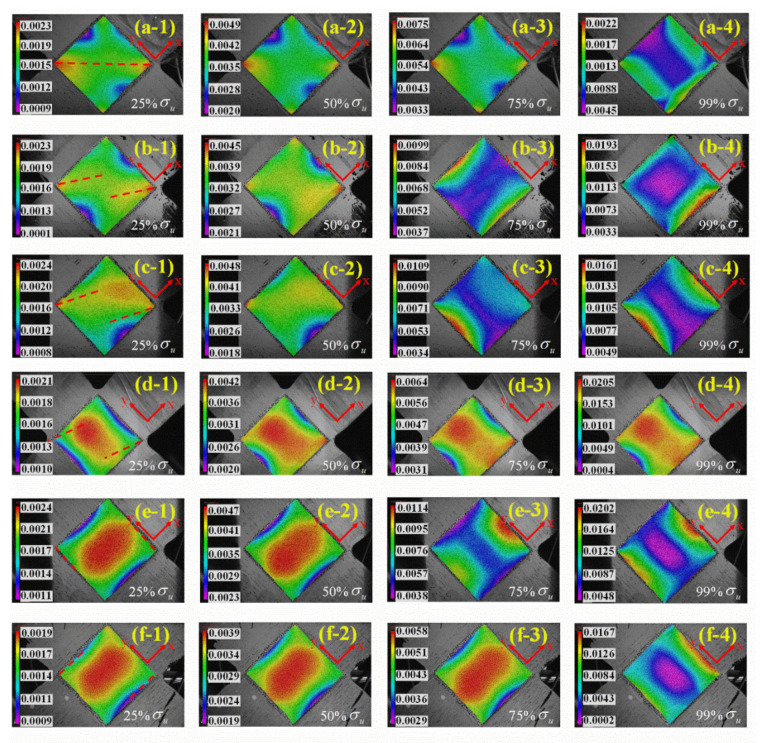
Maximum principal strain distribution of the effective test region with the loading ratio in X and Y axes by (**a**) 3:1, (**b**) 2:1, (**c**) 1:1, (**d**) 0.67:1, (**e**) 1:0, (**f**) 0:1.

The strain fields of the central testing region of the cruciform specimens during the loading process are captured and analyzed by the DIC technique, as shown in Figure 11. The maximum strain mainly occurs in the effective test region from the contour diagram, which verifies the effectiveness of the cruciform specimen designed in this paper. The stress concentration surfaces under different loading ratios are observed via the DIC technique, as marked in Figure 11(a1–f1).

When the loading ratio changes from 3:1 to 0.67:1, the angle between the stress concentration surface and the X-axis gradually decreases from 45°. The angles are 90° and 0° when the loading ratios are 1:0 and 0:1, respectively. The varying angles between the stress concentration surface and the loading axis indicate that different loading ratios will result in different failure surface positions and failure modes, which will be discussed in detail in the next subsection.

### 4.3. Failure Analysis

**Figure 12:**
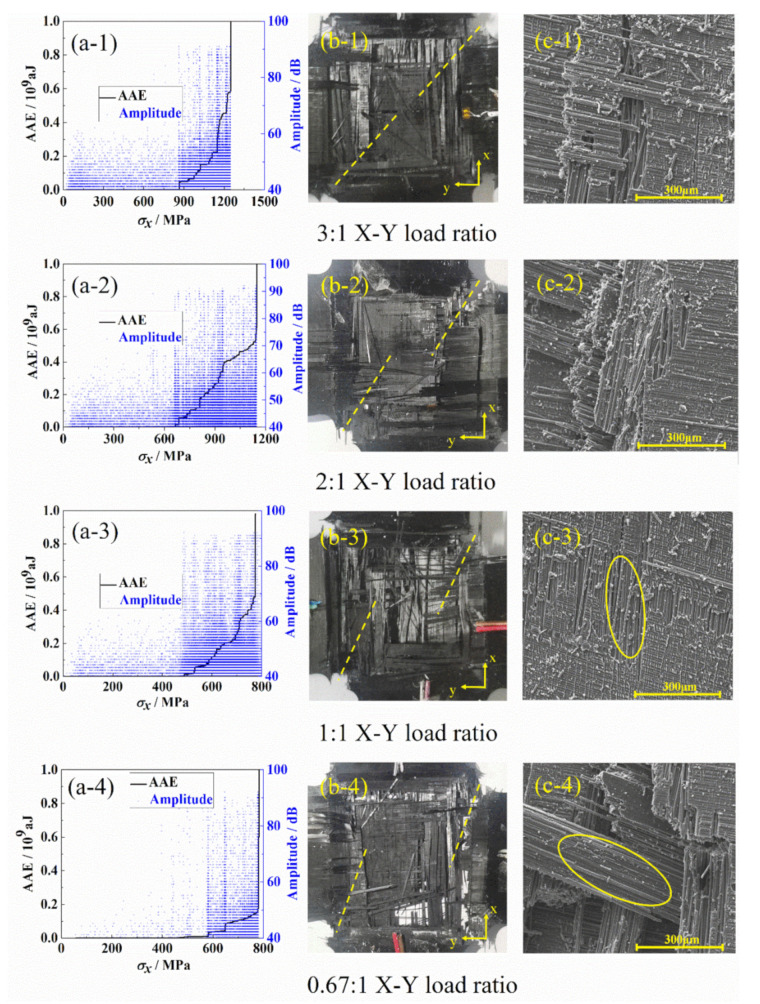
Failure mode and morphology analysis by (**a**) acoustic emission, (**b**) macroscopic damage morphology, and (**c**) SEM images of bare fiber groups.

Different failure modes during the loading process can be identified by the acoustic emission technique because different failure modes occurring in composites will produce different amplitudes of sound. When the amplitude is between 40 and 60 dB, the damage mode is matrix cracking. When it is between 60 and 80 dB, the damage mode is mainly interlayer damage because the matrix friction during the delamination will cause higher acoustic emission signal amplitude than matrix cracking. When the amplitude is higher than 80 dB, the damage mode is fiber fracture. The accumulated absolute energy (AAE) reflects the damage degree of the specimen under biaxial tension. From the acoustic emission results in Figure 12a, when the AAE is below $10^{7}aJ$, a small amount of matrix cracking will occur in the specimen. Corresponding to the stress–strain curves measured by the strain gauges (Figure 9a), it can be seen that the X-axial stress is linearly increasing under 900 MPa. When the AAE is greater than $10^{7}aJ$, the matrix cracking, delamination, debonding, and fiber fracture exist in the specimen simultaneously. Correspondingly, in Figure 9a, the slope of the line begins to change when the stress is larger than 900 MPa. The results show that the initial inflection point in the stress–strain curve is consistent with the inflection point of the amplitude and the AAE curve, which indicates that the biaxial test platform constructed in this paper can accurately capture the change of the failure mode during the biaxial tension.

With the decrease in loading ratio, the growth rate of AAE decreases gradually before biaxial tensile fracture, and the specimen tends to break suddenly at the ultimate stress. In the microscopic images of typical areas obtained by scanning electron microscope (SEM), it is common to show a large area of bare fiber groups, as marked in Figure 12c. According to the macroscopic damage morphology of the biaxial tensile specimens in Figure 12b, with the decrease in loading ratio, the included angle between the failure surface and the X-axis in the effective test area gradually decreases. This is consistent with the distribution trend of stress concentration surface observed by DIC.

### 4.4. Biaxial Failure Envelope

**Figure 13:**
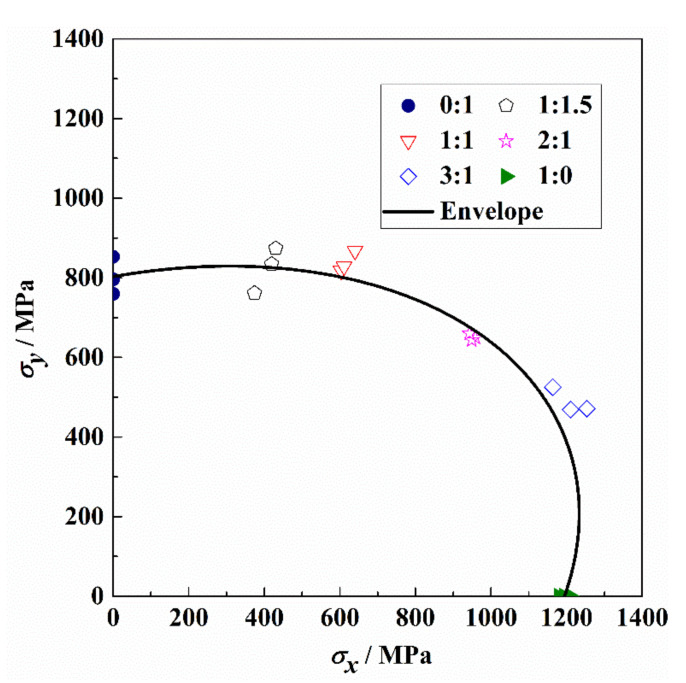
The tension–tension failure envelope.

From the uniaxial and biaxial tension test results of the cruciform specimens in Section 4.1, the coincidence between each loading ratio shows the repeatability of the experimental procedure. The Norris criterion is utilized to fit the biaxial failure envelope [38]:

(7) $$F_{11}\sigma_{x}^{2}+F_{22}\sigma_{y}^{2}+2F_{12}\sigma_{x}\sigma_{y}=1$$

where $F_{11}=\frac{1}{X_{t}X_{t}},F_{22}=\frac{1}{Y_{t}Y_{t}},F_{12}=-\frac{1}{2X_{t}Y_{t}}$, $X_{t}$ is the tensile strength in the X-axis direction of the [90°/0°_2_]_2s_ laminate, and $Y_{t}$ is the tensile strength in the X-axis direction of the laminate. The biaxial tension–tension failure envelope in the first quadrant is shown in Figure 13. It can be seen from the failure envelope curve of the biaxial tensile, that the strength envelope is convex in the first quadrant. Compared with the X-axis uniaxial tensile, the ultimate tensile strength of the X-axis first increases and then decreases with the decrease in the X-Y load ratio under the biaxial tension. Similarly, the ultimate tensile strength of the Y-axis under the biaxial tension can be found to increase first and then decrease with the increase in the X-Y load ratio compared to the Y-axis uniaxial tensile. This biaxial strengthening effect has been reported in the studies of biaxial tension of glass/epoxy composites [39,40]. The mechanism can be identified based on the shape of the highest-order failure theory predictions [41].

## 5. Conclusions

To investigate the mechanical properties of carbon-fiber-reinforced polymer (CFRP) composites under biaxial load, the design criteria of the cruciform specimen are developed by introducing the Hashin criterion. A weighted design function is proposed to characterize the stress concentration factor and the degree of stress uniformity of the cruciform specimen. The effect of geometric parameters on the stress distribution in the central test region is discussed using finite element analysis. The embedded continuous laying method is proposed to free the specimens from initial defects caused by the milling process. The high process quality of the manufactured cruciform specimens is verified using an ultrasonic C-scanning test. The biaxial test platform, which consists of the biaxial loading test system, digital image correlation (DIC) system, strain electrical measurement system, and acoustic emission detection system, is constructed. The biaxial tensile tests under different loading ratios in the X and Y direction are carried out, and the variations of strain fields and the acoustic emission signals during the loading are monitored. The biaxial failure mechanism of CFRP is revealed by the macro/microscopic observation method. The tension–tension failure envelope is established based on the biaxial test. The results show that:

(1) The biaxial failure occurs in the central effective test region of the cruciform specimen, verifying the effectiveness of the design and manufacturing method proposed in this paper.
(2) Under various biaxial loading ratios, the stress concentration angle observed by DIC decreases with the decrease in the X-Y axial loading ratio. This is consistent with the final failure angle.
(3) With the decrease in the X-Y axial loading ratio, the specimen tends to break instantaneously under the ultimate stress, and it is easier to observe the large-area bare fiber group caused by delamination in the microstructure.

## Figures and Tables

**Table 1:** Geometric parameters of the initial configuration of the cruciform specimen.

Geometric Parameters	Symbol	Values before Optimization
Length of loading arm	L	112.5 mm
Width of loading arm	W	50 mm
Necking angle	α	8°
Distance between necking angle and transition area	$l_{\alpha }$	6 mm
Distance between center of transition arc and center of cruciform specimen	$l_{\mathrm{arc}}$	29 mm
Thickness of the effective test region	$h_{\mathrm{eff}}$	1.5 mm
Radius of transition arc	$r_{\mathrm{arc}}$	5.4 mm
Chamfering of the effective test region	$r_{\mathrm{eff}}$	4 mm
Side length of the effective test region	$l_{\mathrm{eff}}$	36 mm
Side length of central variable thickness region	$l_{\mathrm{var}}$	48 mm

**Table 2:** Material properties of T300/epoxy resin lamina.

*E*_1_/GPa	*E*_2_/GPa	*E*_3_/GPa	*G*_12_/GPa	*G*_13_/GPa	*G*_23_/GPa	*μ*_12_	*μ*_13_	*μ*_23_
125	9.75	9.75	7.5	7.5	5.3	0.318	0.318	0.318

## Data Availability

The raw/processed data required to reproduce these findings cannot be shared at this time due to technical or time limitations but can be made available upon reasonable request to the corresponding author.

## References

[1] Ren M., Zhang X., Huang C., Wang B., Li T. (2019). An Integrated Macro/Micro-Scale Approach for in Situ Evaluation of Matrix Cracking in the Polymer Matrix of Cryogenic Composite Tanks. Compos. Struct..

[2] Huang C., Ren M., Li T., Chang X., Cong J., Lei Y. (2016). Trans-Scale Modeling Framework for Failure Analysis of Cryogenic Composite Tanks. Compos. Part B.

[3] Hamori H., Kumazawa H., Higuchi R., Yokozeki T. (2020). Gas Permeability of CFRP Cross-Ply Laminates with Thin-Ply Barrier Layers under Cryogenic and Biaxial Loading Conditions. Compos. Struct..

[4] Zhang X., Li T., Huang C., Huang Q., Ren M., Wang B. (2021). Determining the Fiber/Matrix Interfacial Shear Strength under Cryogenic Conditions by Statistical Inversion. Polym. Compos..

[5] Chen J., Wan L., Ismail Y., Ye J., Yang D. (2021). A Micromechanics and Machine Learning Coupled Approach for Failure Prediction of Unidirectional CFRP Composites under Triaxial Loading: A Preliminary Study. Compos. Struct..

[6] Liao B., Jia L., Zhou J., Lei H., Gao R., Lin Y., Fang D. (2020). An Explicit–Implicit Combined Model for Predicting Residual Strength of Composite Cylinders Subjected to Low Velocity Impact. Compos. Struct..

[7] Antoniou A.E., Van Hemelrijck D., Philippidis T.P. (2010). Failure Prediction for a Glass/Epoxy Cruciform Specimen under Static Biaxial Loading. Compos. Sci. Technol..

[8] Prabhu G., Katakam V., Sridharan V.S., Idapalapati S. (2019). Uniaxial Tensile Failure of Multi-Core Asymmetric Sandwich Composite Structures with Bonded Repair. Compos. Struct..

[9] Na W., Lee G., Sung M., Han H.N., Yu W.R. (2017). Prediction of the Tensile Strength of Unidirectional Carbon Fiber Composites Considering the Interfacial Shear Strength. Compos. Struct..

[10] Bhuiyan F.H., Sanei S.H.R., Fertig R.S. (2020). Predicting Variability in Transverse Effective Elastic Moduli and Failure Initiation Strengths in UD Composite Microstructures Due to Randomness in Fiber Location and Morphology. Compos. Struct..

[11] Garulli T., Catapano A., Fanteria D., Huang W., Jumel J., Martin E. (2020). Experimental Assessment of Fully-Uncoupled Multi-Directional Specimens for Mode I Delamination Tests. Compos. Sci. Technol..

[12] Liu G., Zhang L., Guo L., Liao F., Zheng T., Zhong S. (2019). Multi-Scale Progressive Failure Simulation of 3D Woven Composites under Uniaxial Tension. Compos. Struct..

[13] Hu Z., Chen M.H., Zu L., Jia X., Shen A., Yang Q., Xu K. (2021). Investigation on Failure Behaviors of 70 MPa Type IV Carbon Fiber Overwound Hydrogen Storage Vessels. Compos. Struct..

[14] Nebe M., Asijee T.J., Braun C., van Campen J.M.J.F., Walther F. (2020). Experimental and Analytical Analysis on the Stacking Sequence of Composite Pressure Vessels. Compos. Struct..

[15] Wu Q., Wang Q., Ren S., Zu L., Zhang Q., Zhang G. (2021). Perforation of Carbon Fiber-Wound Composite Cylinders Struck by Hemispherical and Conical-Nosed Impactors. Compos. Struct..

[16] Wang Q., Li T., Wang B., Liu C., Huang Q., Ren M. (2020). Prediction of Void Growth and Fiber Volume Fraction Based on Filament Winding Process Mechanics. Compos. Struct..

[17] Liu G., Gao H., Wei G., Ma Y. (2019). A Novel Structure Design of Braided Composite Pressure Vessel and Its Mechanical Analysis. J. Text. Inst..

[18] Bai J., Wang Z., Sobey A., Shenoi A. (2021). Micromechanical Model for Rapid Prediction of Plain Weave Fabric Composite Strengths under Biaxial Tension. Compos. Struct..

[19] He R., Sun X., Wu Y., Tang G., Carvelli V. (2021). Biaxial Tearing Properties of Woven Coated Fabrics Using Digital Image Correlation. Compos. Struct..

[20] Skinner T., Datta S., Chattopadhyay A., Hall A. (2019). Fatigue Damage Behavior in Carbon Fiber Polymer Composites under Biaxial Loading. Compos. B Eng..

[21] Greco F., Leonetti L., Medaglia C.M., Penna R., Pranno A. (2018). Nonlinear Compressive Failure Analysis of Biaxially Loaded Fiber Reinforced Materials. Compos. B Eng..

[22] Du K., Huang S., Shi M., Li L., Huang H., Zhang S., Zheng W., Yuan X. (2021). Effects of Biaxial Tensile Mechanical Properties and Non-Integer Exponent on Description Accuracy of Anisotropic Yield Behavior. Mater. Des..

[23] Rashidi A., Milani A.S. (2018). A Multi-Step Biaxial Bias Extension Test for Wrinkling/de-Wrinkling Characterization of Woven Fabrics: Towards Optimum Forming Design Guidelines. Mater. Des..

[24] Daniel I., Whitney J., Pipes R. (1983). Experimental Mechanics of Fiber Reinforced Composite Materials. Exp. Tech..

[25] Smits A., Van Hemelrijck D., Philippidis T.P., Cardon A. (2006). Design of a Cruciform Specimen for Biaxial Testing of Fibre Reinforced Composite Laminates. Compos. Sci. Technol..

[26] Moreno M.C.S., Cela J.J.L. (2011). Failure Envelope under Biaxial Tensile Loading for Chopped Glass-Reinforced Polyester Composites. Compos. Sci. Technol..

[27] Welsh J.S., Adams D.F. (2002). An Experimental Investigation of the Biaxial Strength of IM6/3501-6 Carbon/Epoxy Cross-Ply Laminates Using Cruciform Specimens. Compos. Part A Appl. Sci. Manuf..

[28] Makris A., Vandenbergh T., Ramault C., Van Hemelrijck D., Lamkanfi E., Van Paepegem W. (2010). Shape Optimisation of a Biaxially Loaded Cruciform Specimen. Polym. Test..

[29] Gower M.R.L., Shaw R.M. (2010). Towards a Planar Cruciform Specimen for Biaxial Characterisation of Polymer Matrix Composites. Applied Mechanics and Materials.

[30] Gan K.W., Allegri G., Hallett S.R. (2016). A Simplified Layered Beam Approach for Predicting Ply Drop Delamination in Thick Composite Laminates. Mater. Des..

[31] Vidyashankar B.R., Murty A.V.K. (2001). Analysis of Laminates with Ply Drops. Compos. Sci. Technol..

[32] Lamkanfi E., Van Paepegem W., Degrieck J., Ramault C., Makris A., Van Hemelrijck D. (2010). Strain Distribution in Cruciform Specimens Subjected to Biaxial Loading Conditions. Part 2: Influence of Geometrical Discontinuities. Polym. Test..

[33] Pagano N.J., Pipes R.B. (1970). Interlaminar Stresses in Composite Laminates under Uniform Axial Extension(Composite Laminates under Uniform Axial Strain, Determining Interlaminar Stresses and Displacements by Finite Difference Techniques). J. Compos. Mater..

[34] Correa E., Barroso A., Pérez M.D., París F. (2017). Design for a Cruciform Coupon Used for Tensile Biaxial Transverse Tests on Composite Materials. Compos. Sci. Technol..

[35] Hashin Z., Rotem A. (1973). A Fatigue Failure Criterion for Fiber Reinforced Materials. J. Compos. Mater..

[36] Wang Q., Li T., Yang X., Wang K., Wang B., Ren M. (2021). Prediction and Compensation of Process-Induced Distortions for L-Shaped 3D Woven Composites. Compos. Part A Appl. Sci. Manuf..

[37] Wang Q., Li T., Yang X., Huang Q., Wang B., Ren M. (2020). Multiscale Numerical and Experimental Investigation into the Evolution of Process-Induced Residual Strain/Stress in 3D Woven Composite. Compos. Part A Appl. Sci. Manuf..

[38] Norris C. (1962). Strength of Orthotropic Materials Subjected to Combined Stresses.

[39] Kobeissi A., Rahme P., Leotoing L., Guines D. (2020). Strength Characterization of Glass/Epoxy Plain Weave Composite under Different Biaxial Loading Ratios. J. Compos. Mater..

[40] Welsh J.S., Mayes J.S., Key C.T., McLaughlin R.N. (2004). Comparison of MCT Failure Prediction Techniques and Experimental Verification for Biaxially Loaded Glass Fabric-Reinforced Composite Laminates. J. Compos. Mater..

[41] Soden P.D., Hinton M.J., Kaddour A.S. (1998). A Comparison of the Predictive Capabilities of Current Failure Theories for Composite Laminates. Compos. Sci. Technol..

